# The Effective Charge of Low-Fouling Polybetaine Brushes

**DOI:** 10.1021/acs.langmuir.5c00759

**Published:** 2025-06-10

**Authors:** Alina Pilipenco, Michala Forinová, Zulfiya Černochová, Zdeňka Kolská, Ladislav Fekete, Hana Vaisocherová-Lísalová, Milan Houska

**Affiliations:** † 86889 FZU—Institute of Physics of the Czech Academy of Sciences, Na Slovance 1999/2, Prague 180 00, Czech Republic; ‡ Institute of Physics, Faculty of Mathematics and Physics, Charles University, Ke Karlovu 3, Prague 121 16, Czech Republic; § Institute of Macromolecular Chemistry CAS, Heyrovského nám. 2, Prague 16206, Czech Republic; ∥ Faculty of Science, J. E. Purkyně University in Ústí nad Labem, Pasteurova 15, Ústí nad Labem 400 96, Czech Republic

## Abstract

Polybetaine nanobrushes are widely used as inert platforms for label-free biosensing due to their resistance to nonspecific interactions. Despite being considered cationic or electrically neutral, polybetaines can exhibit a negative zeta potential (ZP) at pHs above their isoelectric point (pI). To clarify whether negative zeta potential effectively contributes to surface interactions, we examined three types of nanobrushes deposited on a planar gold substrate: two polybetaines: poly­(carboxybetaine methacrylamide) (pCBMAA) and poly­(sulfobetaine methacrylamide) (pSBMAA) and hydrophilic poly­[*N*-(2-hydroxypropyl) methacrylamide] (pHPMAA), which carries no ionic group. All three brushes exhibit a well-defined pI and negative surface ZP at pHs above their pI. The pH dependence of the interactions of these brushes with anionic dextran sulfate (DS) and cationic poly­[(*N*-trimethylammonium)­ethyl methacrylate] (PTMAEMA) was monitored by infrared reflection spectroscopies (infrared reflection absorption spectroscopy (IRRAS), grazing angle attenuated total reflectance (GAATR)). DS adsorbs to pCBMAA strongly and only weakly to pSBMAA at pHs below their pI but can adsorb slightly to both polybetaines even at pHs above their pI. This is due to the displacement of their carboxylate or sulfo groups from the interaction with the quaternary ammonium cation by the DS sulfate groups. However, DS does not adsorb to pHPMAA at any pH, and PTMAEMA does not adsorb to any of the brushes, regardless of pH. These findings highlight that zeta potential determinations alone may not be sufficient to predict electrostatic interactions as the apparent negative charge does not necessarily translate into a functional surface charge influencing macromolecular interactions.

## Graphical abstract

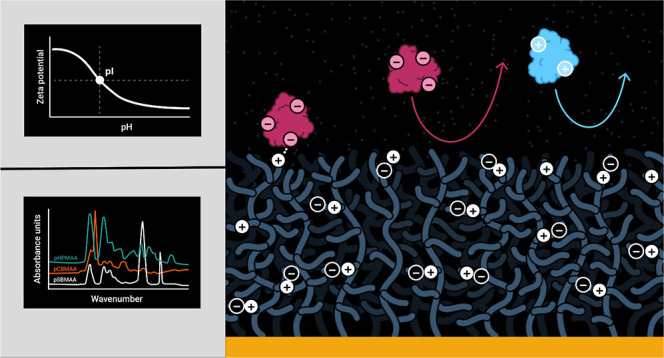

## 1 Introduction

Polybetaine brushes, which are linear polymers with one end of the chain tethered to a solid substrate, have gained a lot of interest due to their unique properties, namely, their resistance to nonspecific interactions with biological media. Polybetaines represent a specific group of zwitterionic polymers because, by definition, in one monomer unit, they contain a pair of a permanent cationic group that bears no hydrogen atom, such as a quaternary ammonium (QA) group and a negatively charged group, such as a carboxylate or sulfo group. It is generally believed that betaines and their analogues can exist in only two ionic forms: zwitterionic (electrically neutral) and cationic, but not anionic.^1–5^

Poly­(carboxybetaine methacrylamide) (pCBMAA) contains a permanent quaternary ammonium cation (QA) and a carboxyl group. Therefore, at pH values lower than its isoelectric point (pI), pCBMAA may become positively charged as carboxylate groups become protonated, and the positive QA charge predominates. At pHs higher than its pI, pCBMAA has a zero net charge as the carboxyl groups are completely dissociated and the charge equilibrium is achieved. Therefore, pCBMAA should not become anionic. Similarly, poly­(sulfobetaine methacrylamide) (pSBMAA), containing a permanent sulfo anion and QA cation groups, is electrically neutral over a wide range of pHs. However, some experimental data, primarily zeta potential measurements, suggest that this may not be the case, and polycarboxybetaines may exhibit anionic behavior at pH values above their isoelectric point (pI).^4,6–9^

The electrostatic interactions between polybetaine brushes with the surrounding medium play a key role in the adhesion phenomena, affecting their performance in many application areas. In biosensing, the excellent resistance of polybetaines to nonspecific adsorption improves the detection accuracy and signal-to-noise ratios of the sensors.^10–12^ Their excellent biocompatibility and low protein adsorption have led to their applications in biomedical devices, including implants and stents, where they reduce immune activation and extend device lifespan.^13–15^ For antibacterial coatings, the hydrated polymer layer acts as a physical and energetic barrier to microbial adhesion, reducing infection risks of medical devices.^16^ In the area of drug delivery and nanotheranostics, the pH- or ionic strength-responsive polymer brushes enable controlled release profiles for targeted therapy.^17,18^ In separation technologies, especially in membranes for ultrafiltration, their antifouling properties help maintain flux and selectivity over extended use.^19^ Finally, in microfluidic devices, polybetaine brushes stabilize flow behavior, reduce clogging, and improve analyte recovery by preventing biofouling; for biolubrication, they reduce friction at soft interfaces, mimicking natural lubrication mechanisms; and they act as environmentally responsive coatings for marine and industrial surfaces.^20–24^

The surface charge of devices coated with polybetaine brushes should be carefully optimized for specific applications, and various factors must be considered. Antifouling polycarboxybetaine platforms intended for biosensing purposes place extreme demands on their resistance to fouling, and the net zero charge of polycarboxybetaines is crucial for their exceptional antifouling properties.^25–27^ However, this condition can be met only at pHs close to the pI and/or above the pI, provided that the polycarboxybetaine retains its charge neutrality.

To be able to detect a specific analyte, the biosensing antifouling platform needs to be functionalized by attaching (ideally covalently) a corresponding biorecognition element (BRE), and such a procedure may further affect its charge state.^27,28^ The most common procedure for covalent attachment of a BRE to polycarboxybetaines is via NHS active esters, which react with the amino groups of the BRE to form an amide bond.^27,29^ This reaction consumes one carboxyl group per amide bond. Moreover, not all of the active esters that remain after the BRE immobilization reaction hydrolyze back to the carboxyl group, so they could nonspecifically react with other amino compounds present and lead to false analytical results.^30^ Thus, in practice, the residual active esters are deactivated by the reaction with an agent carrying amino and carboxyl groups in order to restore the original carboxyl groups, albeit at a different position with respect to the QA cation.

Alternatively, the net charge of the polycarboxybetaine brush can also be tuned by copolymerizing with sulfobetaine methacrylamide^31^ or with a monomer containing a QA group.^4^ Another approach to solving this issue is to optimize and reduce the number of carboxyl groups through copolymerization of the carboxybetaine monomer with another hydrophilic monomer that is sufficiently resistant to fouling but does not have any ionizable group and does not participate in the BRE immobilization reaction, such as poly­[*N*-(2-hydroxypropyl) methacrylamide]. In this way, the number of carboxyl groups can be specifically tuned to a necessary minimum optimal for the required BRE loading while retaining the resistance to fouling.^31^ A yet another approach to optimizing the number of carboxyl groups is the use of a procedure allowing the preparation of hierarchical copolymer structures.^32,33^ While carboxybetaine-based brushes can become positively charged at low pH, leading to increased fouling,^34,35^ they have demonstrated excellent long-term antifouling stability over extended periods and repeated exposures of complex biological samples.^36,37^ Zwitterionic and nonionic brushes also remained structurally stable under varying ionic strengths and drying/rehydration, showing reversible changes without degradation and consistently high antifouling performance.^38^

Determination of the electrokinetic potential at the solid/liquid interface is a powerful tool for characterizing the surface electrical charge. The zeta potential provides valuable information about possible electrostatic interactions that affect the stability and behavior of heterogeneous systems.^39^

Published data on zeta potentials for poly­(carboxybetaine)­s show interesting results. For example, silica nanoparticles coated with pCBMAA brushes with 1 or 5 methylene groups separating QA and carboxyl groups showed nearly the same pI of 8.7 and negative zeta potentials at pH values above their pI. The longer spacer separating the two charged groups resulted in significantly more negative zeta potentials.^7^ The spacer effect has been discussed in several other papers, showing that the longer spacer results in a higher p*K*_a_ for the carboxyl ionization equilibrium.^2,3,7,8,40^

Ramireddy et al.^9^ studied self-assembled micelles of poly­(carboxybetaine acrylamide) alkyl substituted at amide nitrogen. They observed negative zeta potentials above pH 6.2, with the micelles being largest at this pH, and their size decreased at both higher and lower pH values, demonstrating electrostatic repulsion at pH values outside the pI. The authors also prepared analogous polyacrylamides with only a carboxyl or QA group. As expected, the negative zeta potential of the carboxyl analogue almost approached zero with a decreasing pH, and the QA analogue exhibited positive values over the entire pH range from 2 to 10. However, above pH 7, its potential decreased significantly, indicating an apparent pH-dependent behavior.

In another interesting study, Guo et al.^4^ explored pH dependence of the zeta potential of a polymethacrylate brush containing a QA group (pQAMA) and a zwitterionic poly­(sulfobetaine methacrylate) (pSBMA) brush containing QA and sulfo groups. Both brushes were deposited on a silicone support. The polymethacrylate with the QA group exhibited a strong positive charge across the entire pH range from 5 to 10, slowly decreasing with increasing pH, quite similarly to that described in the above paper.^9^ On the other hand, the betaine polymer carrying QA and sulfo groups, which could be expected to be neutral, exhibited a negative potential throughout the same pH range with only a small pH dependence. This suggested that the sulfo group acts as a stronger ion. To create the most neutral and pH-nonresponsive brush, the authors copolymerized the positively charged QAMA with negatively charged SBMA and found that just 2 mol % QAMA was optimal for shifting the pH-dependent zeta potential curve closer to zero. This copolymer had a pI of approximately 6.6 and a zeta potential of +10 mV at pH 5 and −15 mV at pH 10. These copolymers were claimed to be low-fouling (i.e., resistant to nonspecific adsorption from complex biological media), but they are not optimal for biosensing applications because they cannot be as easily functionalized with BREs as pCBMAA and its various copolymers can be.

The behavior described above is not unusual as the surface charge depends on several factors, not just on the degree of ionization of surface groups. The charge at the interface and, therefore, the apparent pI can be considerably affected if ions selectively adsorb from the solution to the surface in a particular way. Many surfaces that do not have ionizable groups can still exhibit a defined pI and a negative ZP.^39^ The negative zeta potential of polybetaines at pH values above their pI may be due to complexation of their permanent cation with the hydroxyl anion in the solution.

Although polybetaines have been shown many times to exhibit minimal electrostatic interactions with their surroundings, it is desirable to investigate whether the zeta potential and its effects are reflected in the behavior of the polybetaine brushes. Useful information about the surface charge can be obtained by investigating the interactions of the surface with specific polyelectrolytes. We have shown earlier that albumin deposited on a surface adsorbs heparin, a heavily sulfated polyanion, at pH values lower than the pI of albumin but not at higher pHs, where albumin is negatively charged.^41,42^ We used this approach to create a defined albumin-heparin molecular multilayer system to modify surfaces of medical devices intended for contact with blood.^42^ The formation of multilayers based on the interaction of a polycarboxybetaine (containing a vinylpyridinium cation) was investigated by Kharlampieva et al.^6^ Electrostatic self-assembly was observed to occur with polyanions at acidic pH when the poly­(carboxybetaine) has a net positive charge, but at higher pH values, where it becomes zwitterionic with balanced charges, no interaction occurs with either polyanions or polycations.

To address this issue for polybetaine brushes, in this study, we investigated three types of brushes deposited on a planar gold substrate. These brushes include two polybetaines: poly­(carboxybetaine methacrylamide) (pCBMAA) and poly­(sulfobetaine methacrylamide) (pSBMAA) and a reference nonionic hydrophilic poly­[*N*-(2-hydroxypropyl) methacrylamide] (pHPMAA). The chemical structures of these polymer brushes are presented in Scheme 1.

**Scheme 1:**
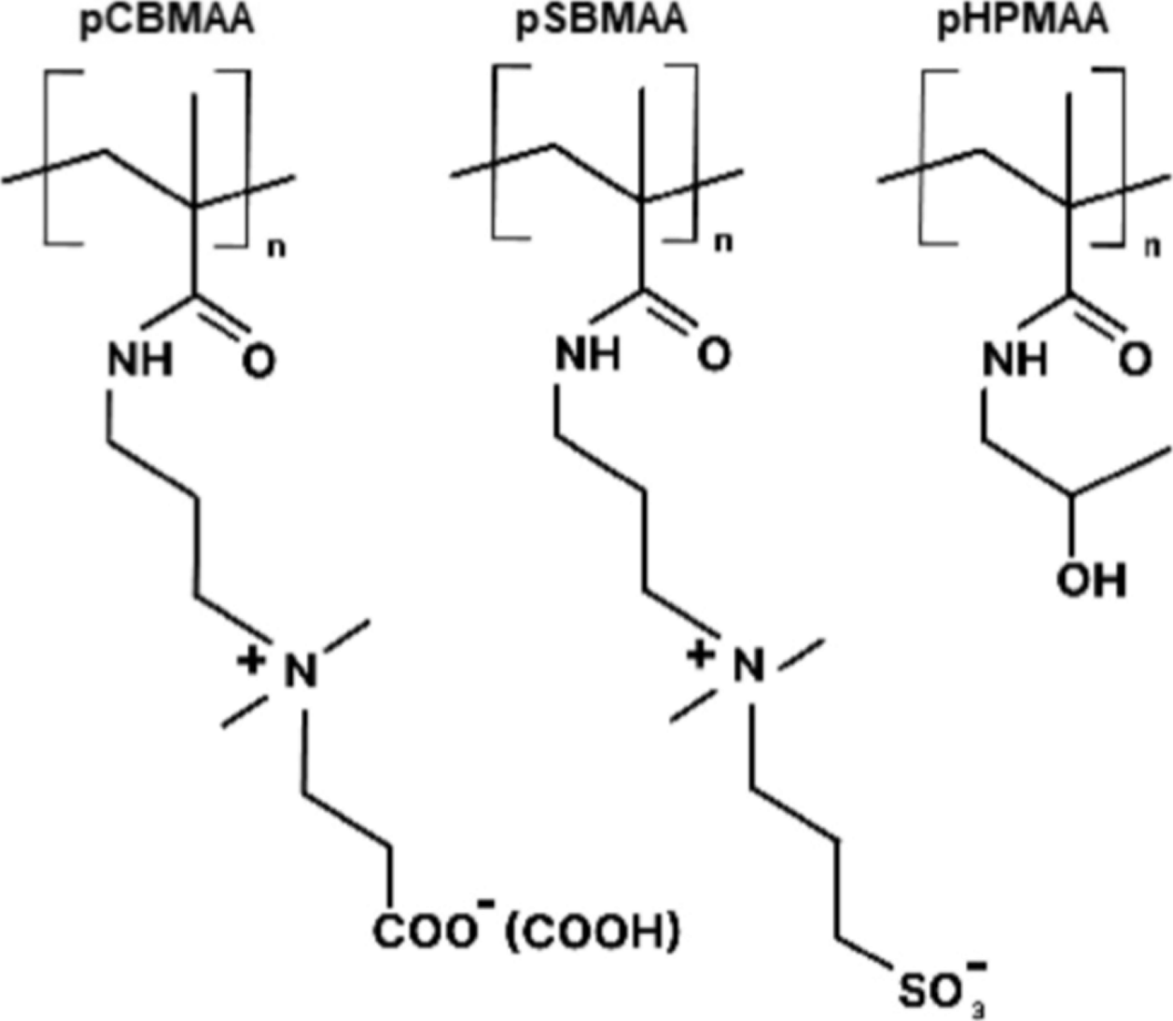
Chemical Structure of Poly­(carboxybetaine methacrylamide) (pCBMAA), Poly­(sulfobetaine methacrylamide) (pSBMAA), and Poly­[*N*-(2-hydroxypropyl) methacrylamide] (pHPMAA) Employed in This Study

This study aims to investigate how the charge of the brushes is manifested during interactions with specific negatively and positively charged polyelectrolytes monitored by infrared spectroscopy and correlated with the determination of the zeta potential. The pH dependence of the surface charge of the brushes was elucidated using two different zeta potential techniques, the electrokinetic and surface zeta potential techniques and two infrared reflection techniques: the infrared reflection–absorption spectroscopy and grazing angle attenuated total reflectance spectroscopy. Infrared spectroscopy was used to monitor the pH dependence of the interactions of the aforementioned brushes with two different polyelectrolytes: polyanionic polymer dextran sulfate (DS) and polycationic poly­[(*N*-trimethylammonium)­ethyl methacrylate] (PTMAEMA).

## 2 Experimental Section

### 2.1 Materials

Ultrapure water (18.0 MΩ·cm, Milli-Q, Merck, Germany) was used for the aqueous solution preparation and rinsing steps. Sodium chloride (NaCl), 1,4,8,11-tetramethyl-1,4,8,11-tetraazacyclotetradecane (Me_4_Cyclam, 98%), copper­(I) chloride (CuCl, ≥99.995%), copper­(II) chloride (CuCl_2_, 99.999%), and methanol (≥99.9%) were obtained from Merck, Czech Republic. ω-Mercaptoundecyl bromoisobutyrate was purchased from ProChimia, Poland. Carboxybetaine methacrylamide (CBMAA), *N*-(2-hydroxypropyl), methacrylamide (HPMAA), and sulfobetaine methacrylamide (SBMAA) monomers were sourced from Specific Polymers, France. Potassium chloride (KCl), sodium hydroxide (NaOH), citric acid, hydrochloric acid (HCl), and dextran sulfate sodium salt (molecular weight 5000) from *Leuconostoc spp.* were supplied by Merck, Germany. Poly­(trimethylammoniumethylmethacrylic chloride) was obtained from Scientific Polymer Products, USA. Ethanol (96%) and methanol (99.8%, p.a.) were purchased from Penta, Czech Republic, while ethanol (99.8%, UV spectroscopy grade) was from Lachner, Czech Republic. *N*-Ethyl-*N*′-(3-(dimethylamino)­propyl) carbodiimide hydrochloride (EDC), *N*-hydroxysuccinimide (NHS), and ethanolamine hydrochloride were obtained from Cytiva, Sweden. Glycine was sourced from Merck, Germany. 2-(2-Aminoethoxy)­acetic acid (AEAA) was purchased from VWR International, Czech Republic.

### 2.2 Preparation of Polymer Brushes

Polymer brush coatings were synthesized via surface-initiated atom transfer radical polymerization (SI-ATRP) using a specialized polymerization setup developed in-house, as described previously.^37^ Briefly, gold-coated chips (size 20 × 10 mm) were cleaned with UV-ozone, rinsed with ultrapure water and ethanol, and immersed in a 0.1 mM solution of ω-mercaptoundecyl bromoisobutyrate in ethanol for 48 h to create a self-assembled monolayer on the surface. Degassed methanol and ultrapure water were used to prepare catalysts (CuCl, CuCl_2_, and Me_4_Cyclam) and monomer solutions under a nitrogen atmosphere. Monomers, including CBMAA, SBMA, and HPMAA, were dissolved in the catalyst solution. The substrates, precoated with the self-assembled monolayer, were placed in the reactor, and polymerization was carried out at room temperature for 2 h, after which the substrates were rinsed with ultrapure water. They were stored in phosphate-buffered saline (PBS) for 1 day and then transferred to ultrapure water at 6 °C until later use.

### 2.3 Modification of pCBMAA

pCBMAA brushes modified with glycine, (2-aminoethoxy)­acetic acid, and ethanolamine were prepared using a standard EDC/NHS activation procedure described elsewhere.^30^

### 2.4 Surface Zeta Potential Determination by Electrokinetic Analysis (EK Method)

The surface zeta potential (ZP) of the nanobrushes on gold planar chips was determined using an Electrokinetic Analyzer SurPASS (Anton Paar, Austria). The samples were placed in the adjustable gap cell in contact with an electrolyte (0.001 M KCl in distilled water) at room temperature. For each measurement, a pair of chips with the same coating was fixed on two sample holders with a cross-section of 20 × 10 mm and a cell gap of 100 μm. For the measurement of the pH dependence of ZP, the samples were titrated with 0.05 M HCl or 0.05 M NaOH solutions. The streaming current method and the Helmholtz–Smoluchowski equation were used. The fitting parameters of titration data are shown in Table S1.

### 2.5 Surface Zeta Potential Determination by Analysis of Electrophoretic Mobility by Displacement from the Surface (EPM Method)

The surface zeta potential of the nanobrushes on gold planar chips was measured using DTS1235 as a tracer particles water solution and a Zetasizer NanoZS Instrument, model ZEN3600 (Malvern Instruments, Malvern, UK), at a scattering angle of θ = 13°. The data were processed with the Malvern software.^43,44^ A Malvern ZEN1020 surface zeta potential cell was used, consisting of a sample barrel with an adjustable height, where the sample was placed on a holder and held between two palladium electrodes. A series of measurements were then performed in the surface zeta potential cell at 25 °C, with the position of the measurement controlled by adjusting the height of the sample holder. For the pH dependence of ZP, the pH of the tracer particle solutions was adjusted using 0.1 M citric acid and 3 M NaOH solutions at the following values: 8.5, 7.0, 5.5, 4.7, 4.0, and 3.0. The brushes were equilibrated 60 min between the measurements. The data were analyzed using the Smoluchowski model.^45^ The obtained titration curves were fitted (Table S1) with a sigmoidal function, which is described by the following equation:

$$y=\frac{A_{1}+\left(A_{2}-A_{1}\right)}{\left(1+10^{\left(\left(\log x_{0}-x\right)*p\right)}\right)}$$

where *A*_1_ = −64.88, *A*_2_ = 32.64, log *x*_0_ = 4.836, and *p* = −0.90909.

### 2.6 Infrared Spectroscopy

All spectra were measured using a Nicolet iS50 spectrometer, ThermoFisher Scientific, USA. The spectrometer was equipped for infrared scanning reflection–absorption spectroscopy (IRRAS) of dry samples with a Specular Apertured Grazing Angle (Smart SAGA) accessory (grazing angle 80°, resolution 4 cm^–1^, aperture 8 mm, 200 scans). The grazing angle attenuated total reflectance (GAATR) spectra were measured using the VariGATR accessory, Harrick Scientific Products, USA (incident beam angle 63°, resolution 4 cm^–1^, 200 scans). The GAATR spectra were measured by placing the wet chip on the Ge ATR prism with a drop of water adjusted to the desired pH and gently pressed until a constant water background signal in the layer between the chip and the prism was established. The reference water background spectrum was measured using an uncoated chip.

### 2.7 Determination of pH Dependence of DS and PTMAEMA Interaction with pCBMAA, pSBMAA, and pHPMAA by Infrared Spectroscopy

The chips coated with pCBMAA, pSBMAA, or pHPMAA brushes were immersed into the aqueous solution of 3 mg/mL DS or PTMAEMA for 20 min with gentle stirring. The pH of the solution was adjusted before starting the experiment by the addition of HCl or NaOH and then continuously monitored. After 20 min, the chips were taken out of the solution and thoroughly rinsed with water adjusted to the same pH as that of the DS or PTMEMA solutions. Then, the chips were dried with a stream of nitrogen and measured immediately. This procedure ensures that DS or PTMAEMA adsorbed irreversibly on the brush at a given pH remains on the surface and can be detected. Both DS and PTMAEMA have distinct characteristic bands in the IRRAS spectrum, enabling their sensitive detection. The bands of the ionized carboxyl groups at 1611 cm^–1^ and protonated carboxyl groups at 1722 cm^–1^ of pCBMAA, sulfate group at 1212 cm^–1^ of pSBMAA, sulfo groups at 1266 cm^–1^ of adsorbed DS, and ester groups of PTMAEMA at 1730 cm^–1^ and 1165 cm^–1^ were monitored by IRRAS, and their integrated intensities were evaluated.

To measure the GAATR spectra of the wet pCBMAA brush at different pH values, the chip was rinsed in the same way but not dried and measured in the wet state, as described above.

### 2.8 Atomic Force Microscopy

The chips coated with the pCBMAA, pSBMAA, or pHPMAA brush were immersed in water whose pH was adjusted to either pH 3.0 or 8.5 by adding HCl or NaOH. After 20 min, the chips were quickly blown with a stream of nitrogen (without rinsing with water) and immediately measured. All measurements were performed at room temperature on a Bruker Dimension Icon AFM operating in the PeakForce Tapping mode with ScanAsyst-Air probes (spring constant ≈ 0.4 N m^–1^; nominal tip radius ≈ 2 nm).

### 2.9 Spectroscopic Ellipsometry

Ellipsometric measurements were performed using a spectroscopic ellipsometer VASE, J.A. Woollam, Lincoln, USA, ranging from 300 cm^–1^ to 1000 cm^–1^ with a 10 nm step and 70° incident angle. The measurements were taken at room temperature using a custom-made cuvette developed for measurements in liquids. The chip was swollen in water with pH adjusted by the addition of HCl or NaOH to 3 to 8. Data analysis was performed with the WVASE32 software. Two samples were measured in each run. Experimental data were fitted with a single Gaussian oscillator model, with an additional water layer as ambient for the measurements in water.

## 3 Results and Discussion

To assess the surface topography, homogeneity, and stability of the brushes when exposed to pH changes, AFM measurements were performed on pCBMAA, pSBMAA and pHPMAA before and after incubation at pH 3.0 and 8.5. The images shown in Figure S1 indicate uniformly coated surfaces without significant patches or defects, confirming good brush coverage. RMS roughness values below 2 nm (Table S2) confirm the presence of smooth stable films with no evidence of significant degrafting or patchiness. These data support the conclusion that each brush maintains its integrity and uniformity over the pH range tested.

The thicknesses of pCBMAA, pSBMAA, and pHPMAA brushes swollen in water at pH 3 to 8.5 were measured by spectroscopic ellipsometry. Figure S2 shows that the thicknesses are in the order of tens of nanometers and are not significantly affected by pH.

Quite recently, the homogeneity of the brushes prepared in a microfluidic stack reactor was verified using a high-resolution infrared microscopy.^46^ In our study, resistance to degrafting was also validated by IRRAS. The IRRAS spectra confirmed the chemical structure of the brushes. Detailed analysis of the spectra and brush interactions with DS and PTMAEMA, as monitored by infrared spectroscopy, is provided in Sections 3.2 and 3.3.

### 3.1 pH Dependence of Zeta Potential for pCBMAA, pSBMAA, and pHPMAA Brushes

#### 3.1.1 pH Dependence of ZP for pCBMAA

pCBMAA contains one ionizable carboxyl group and one quaternary ammonium group, with a permanent positive charge in each monomer unit. The plots of ZP vs pH for pCBMAA, as determined by electrokinetic (EK) and electrophoretic mobility (EPM) methods, are shown in Figure 1. Both methods show a distinct isoelectric point (pI), the EK method at pH 5.6 and the EPM method at pH 4.5. The differences can be caused by the method of titration: during EK titration, the pH of the solution was continuously titrated. For the EPM method, stock solutions with a certain pH value were prepared and used for measurements. Because the layer thickness is on the order of tens of nanometers, it has been found that it is necessary to equilibrate the brush swollen in the tracer particles solution in order to obtain accurate experimental data. The chain length naturally allows for various conformational states, which will buffer during titration experiments. Below the pI, both methods show similar positive ZPs at pH values due to protonation of the carboxyl groups, resulting in a predominantly positive charge from the QA group. The curves differ somewhat above the pI (−20 mV vs −60 mV at pH 7), but both methods show a negative ZP. Due to the betaine structure of pCBMAA, the QA group maintains its positive charge so that the negative charge cannot dominate. Therefore, the positive charge must have been compensated for by the specific adsorption of hydroxyl anions to the surface.

**Figure 1:**
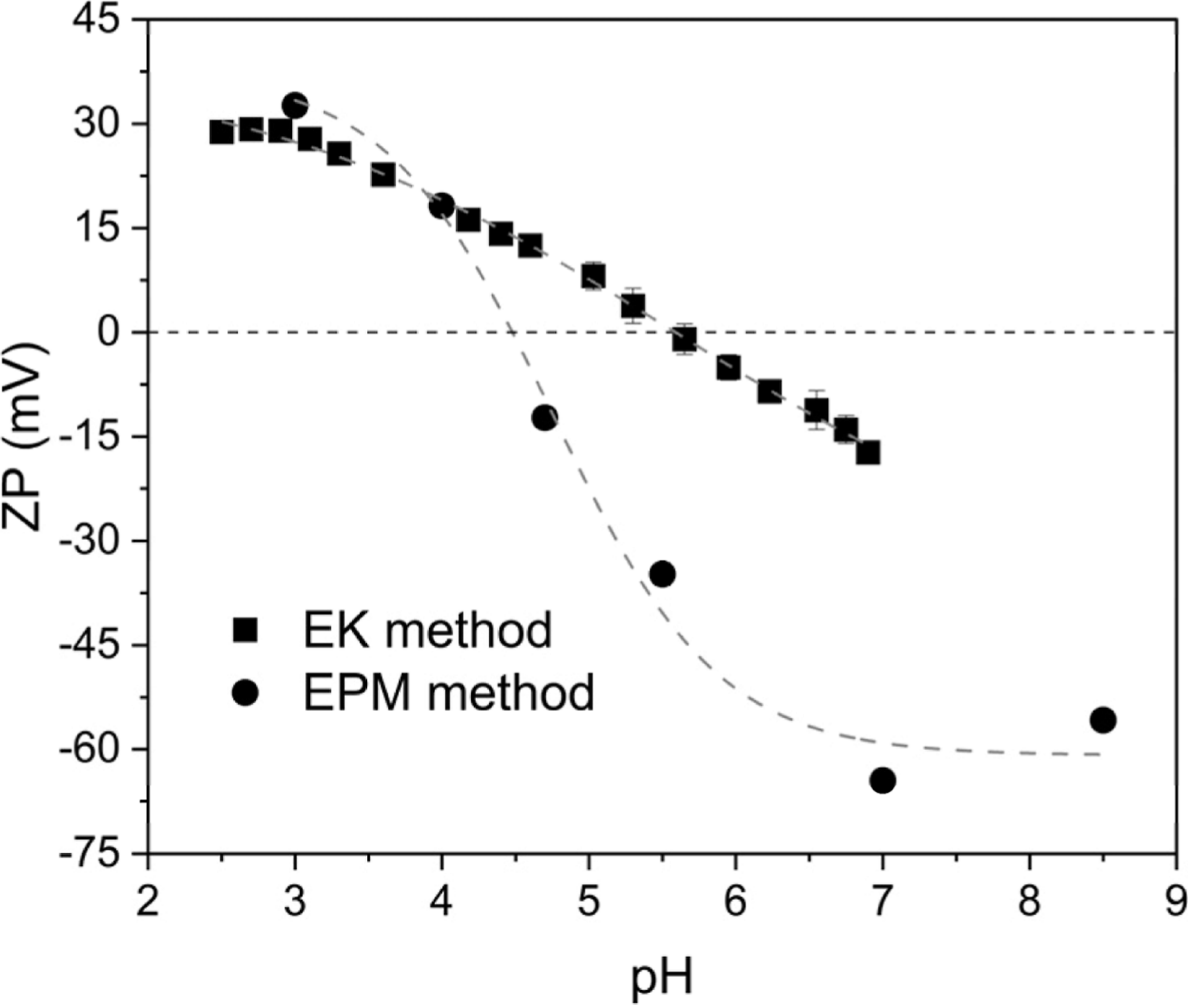
pH dependence of surface zeta potential of the pCBMAA brush measured by the EK method and EPM method.

Based on the behavior of charged particle dispersions, there is a rule that a positive or negative ZP less than 30 mV is not effective enough to repel particles and protect them against aggregation.^39^ Therefore, the question arises as to whether ZP effectively affects the electrostatic interactions of polybetaine brushes and how it is reflected. This issue is of fundamental importance, especially for biosensing label-free applications where even weak nonspecific interactions can play a crucial role.

As discussed in the Introduction section, the functionalization of poly­(carboxybetaine) brushes occurs through their carboxyl groups, leading to changes in the charge balance. To compensate for this, reactions with various reagents are used to deactivate any residual NHS active esters and to replace the consumed carboxyl groups. Therefore, we investigated whether these reactions were reflected in ZP. The reaction conditions were the same as those we commonly use for the functionalization of these brushes.^30^

Table 1 shows the ZP values of the gold chip support and pCBMAA brush measured by the electrokinetic method at pH 6.4, i.e., above its pI, after its EDC/NHS-mediated modification with the appropriate reagents. The gold surface exhibited a high negative ZP value of −76 mV that is effectively reduced by the pCBMAA brush to −11 mV. The modifications did not lead to significant changes in the ZP of the brush, and there was no apparent difference between the pCBMAA brushes modified with carboxy agents and those modified with ethanolamine. Therefore, this indicates that the observed ZP is not primarily driven by the charge balance of the ionizable surface groups. This is further supported by the ZP value of −12.5 mV for pHPMAA, which does not contain any ionizable groups.

**Table 1:** Surface Zeta Potential at pH 6.4 of Gold, Poly­(carboxybetaine methacrylamide) (pCBMAA), and pCBMAA Modified with Glycine, (2-Aminoethoxy)­acetic Acid, and Ethanolamine Determined by the EK Method

	ZP at pH 6.4 [mV]
Au (on glass)	–76
pCBMAA	–11
pCBMAA—glycine	–13
pCBMAA—(2-aminoethoxy)acetic acid	–14
pCBMAA—ethanolamine	–13
pHPMAA	–12

#### 3.1.2 pH Dependence of Zeta Potential for pSBMAA and pHPMAA

pSBMAA contains one anionic sulfo group and one cationic quaternary ammonium group in each monomer unit, both of which retain their charge over a wide range of pH values. pHPMAA is a hydrophilic polymer that does not contain any ionic group. It is well-known for its inertness toward biological media, and it is used for many analytical, biomedical, and pharmaceutical applications. We currently use pHPMAA in copolymers with CBMAA and SBMAA to optimize the number of carboxylate groups in functionalized biosensing platforms.^31^

The plots of ZP vs pH for pSBMAA and pHPMAA, as measured by the EPM method, are shown in Figure 2.

**Figure 2:**
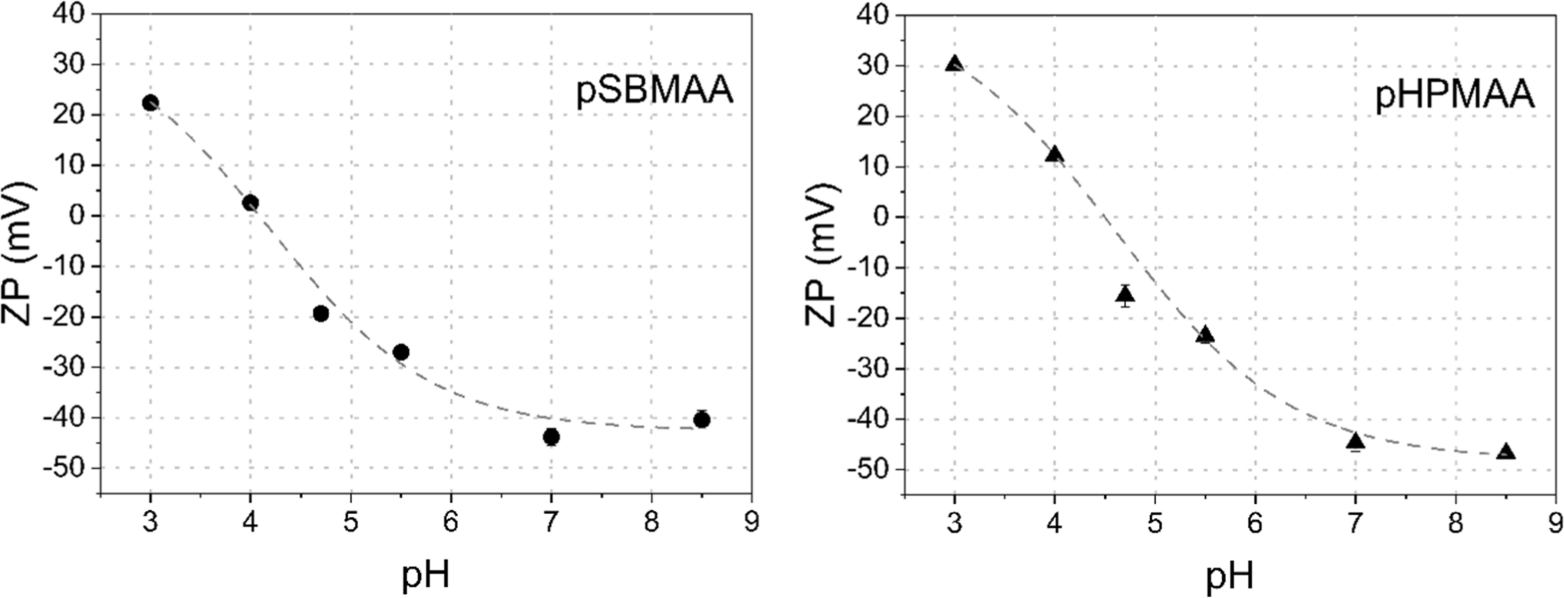
pH dependence of zeta potential of pSBMAA (left) and pHPMAA (right) brushes measured by the EPM method.

Although the structures of the two brushes are fundamentally different, the dependence of their ZP on pH is similar. The pI values of pSBMAA and pHPMAA are 4.0 and 4.3, respectively, and they both exhibit similar negative ZP values close to −40 mV at neutral and slightly alkaline pH values. This is a similar behavior often observed with uncharged hydrophobic polymers, where the surface originates from the preferential adsorption of hydroxide ions in aqueous solutions.^39^

### 3.2 pH Dependence of Interactions of pCBMAA, pSBMAA, and pHPMAA Brushes with DS Evaluated by Infrared Spectroscopy

As discussed above, all three brushes exhibit negative ZP values at pH values above their pI. To investigate whether and how the observed ZP affects the electrostatic interactions of these brushes, we monitored the pH dependence of adsorption of anionic dextran sulfate (DS) and cationic poly­[(*N*-trimethylammonium)­ethyl methacrylate] (PTMAEMA), both strong polyelectrolytes whose charges are independent of pH. The interactions were investigated using reflection infrared spectroscopy, which enables sensitive detection of both agents adsorbed on the above nanobrushes.

Figure 3 shows the IRRAS spectra of all the studied brushes and their major characteristic bands, namely, the two amide bands (Am I: around 1650 cm^–1^, Am II: around 1530 cm^–1^), the two bands of the ionized carboxyl group (COO^–^: 1611 cm^–1^, 1365 cm^–1^), the band of the protonated carboxyl group (COOH: 1722 cm^–1^), and the two bands of the sulfo group (SO_3_^–^: 1212 cm^–1^, 1040 cm^–1^). Figure 4 presents the IRRAS spectra of DS (OSO_3_^–^: 1230 cm^–1^, 1010 cm^–1^) and PTMAEMA (COOR: 1730 cm^–1^, 1165 cm^–1^).

**Figure 3:**
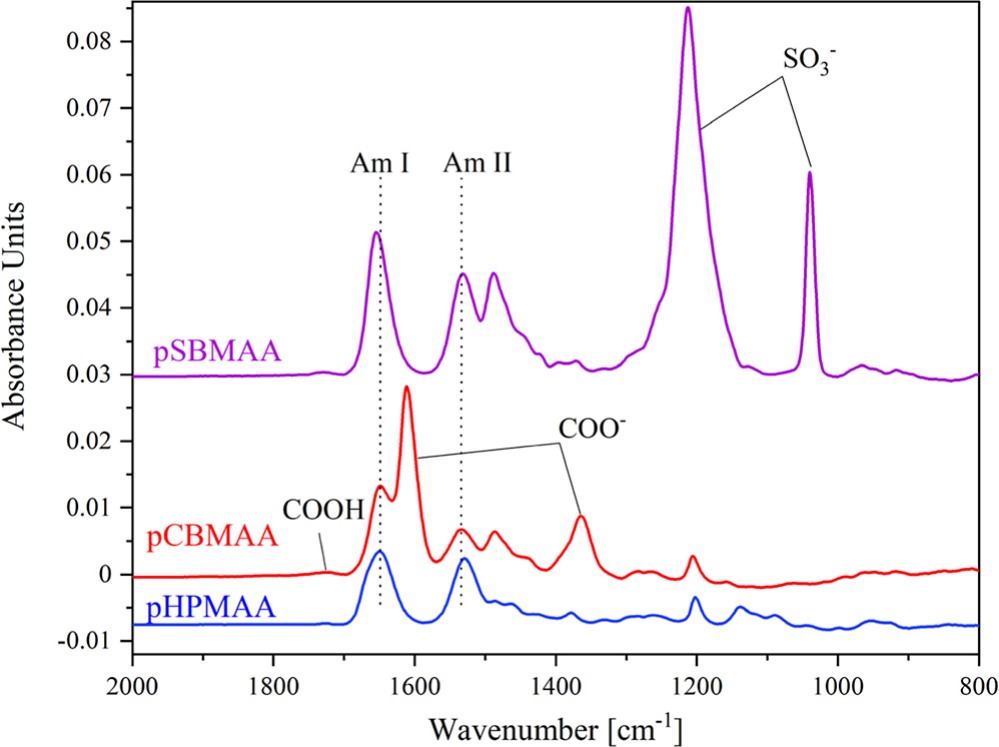
IRRAS spectra of pCBMAA, pSBMAA, and pHPMAA brushes.

**Figure 4:**
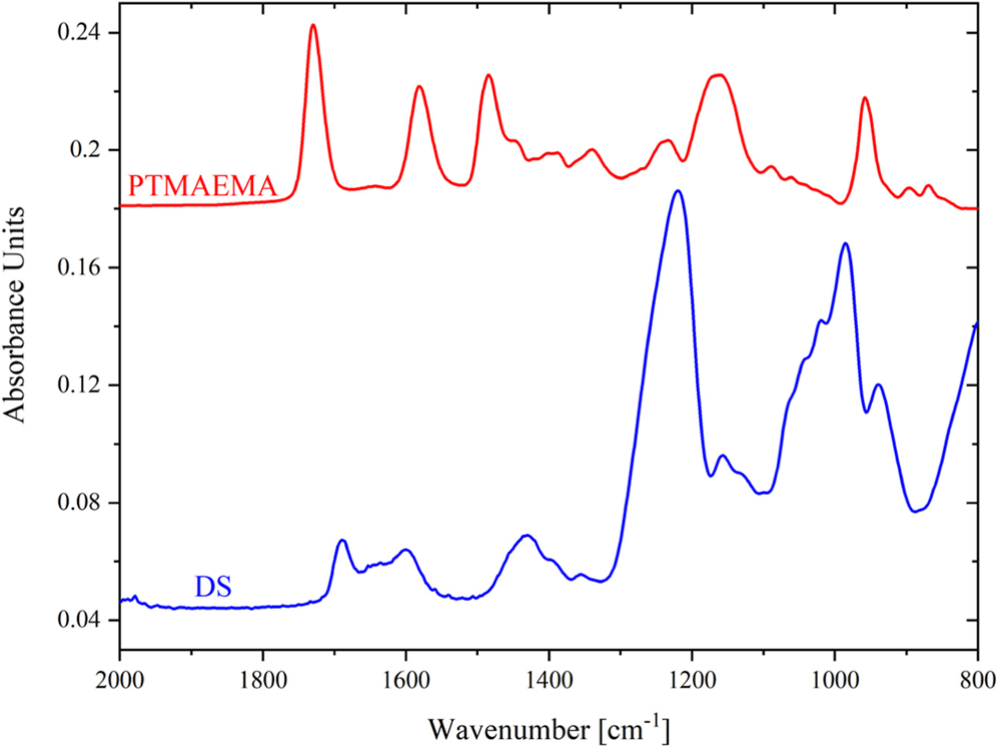
IRRAS spectra of DS and PTMAEMA (a drop of water solution evaporated on a gold chip).

#### 3.2.1 pCBMAA Interactions with DS

The pH dependence of DS adsorption onto the pCBMAA brush is illustrated in the IRRAS spectra in Figures 5 and 6, where the band intensities of the DS sulfate groups and ionized and protonated carboxyl groups are plotted. The spectra clearly demonstrate the appearance of the DS bands at pH values below 5.5. At the same time, the band corresponding to ionized carboxyl groups disappears, and the band for protonated carboxyl groups develops.

**Figure 5:**
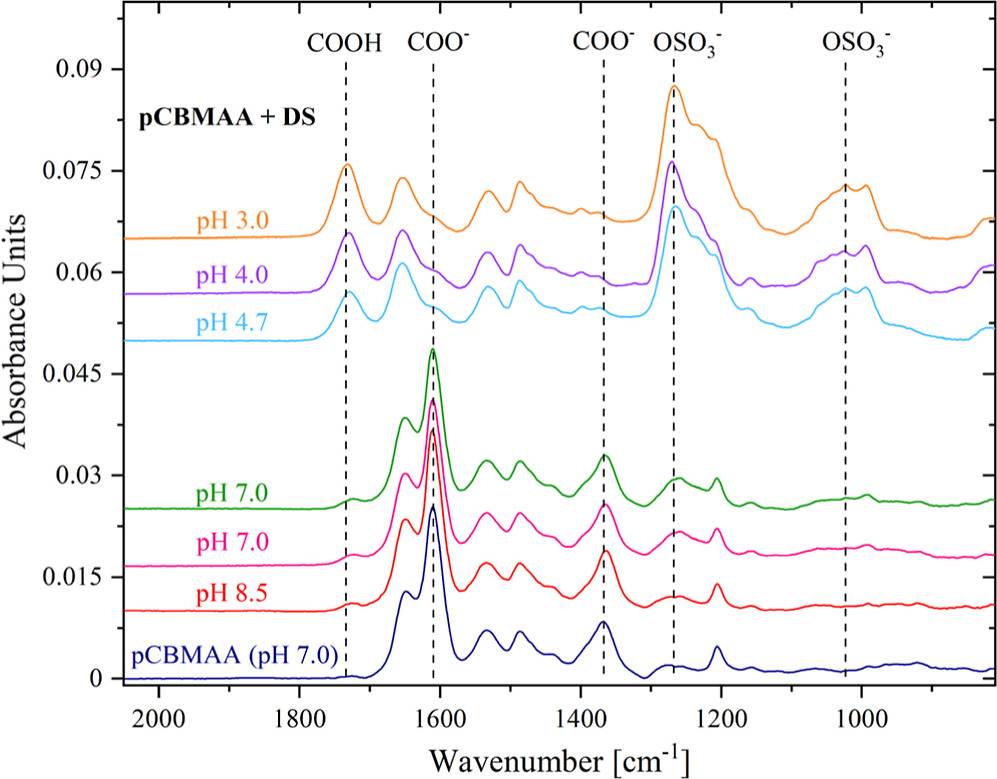
IRRAS spectra of pH dependence of DS adsorption to the pCBMAA brush.

**Figure 6:**
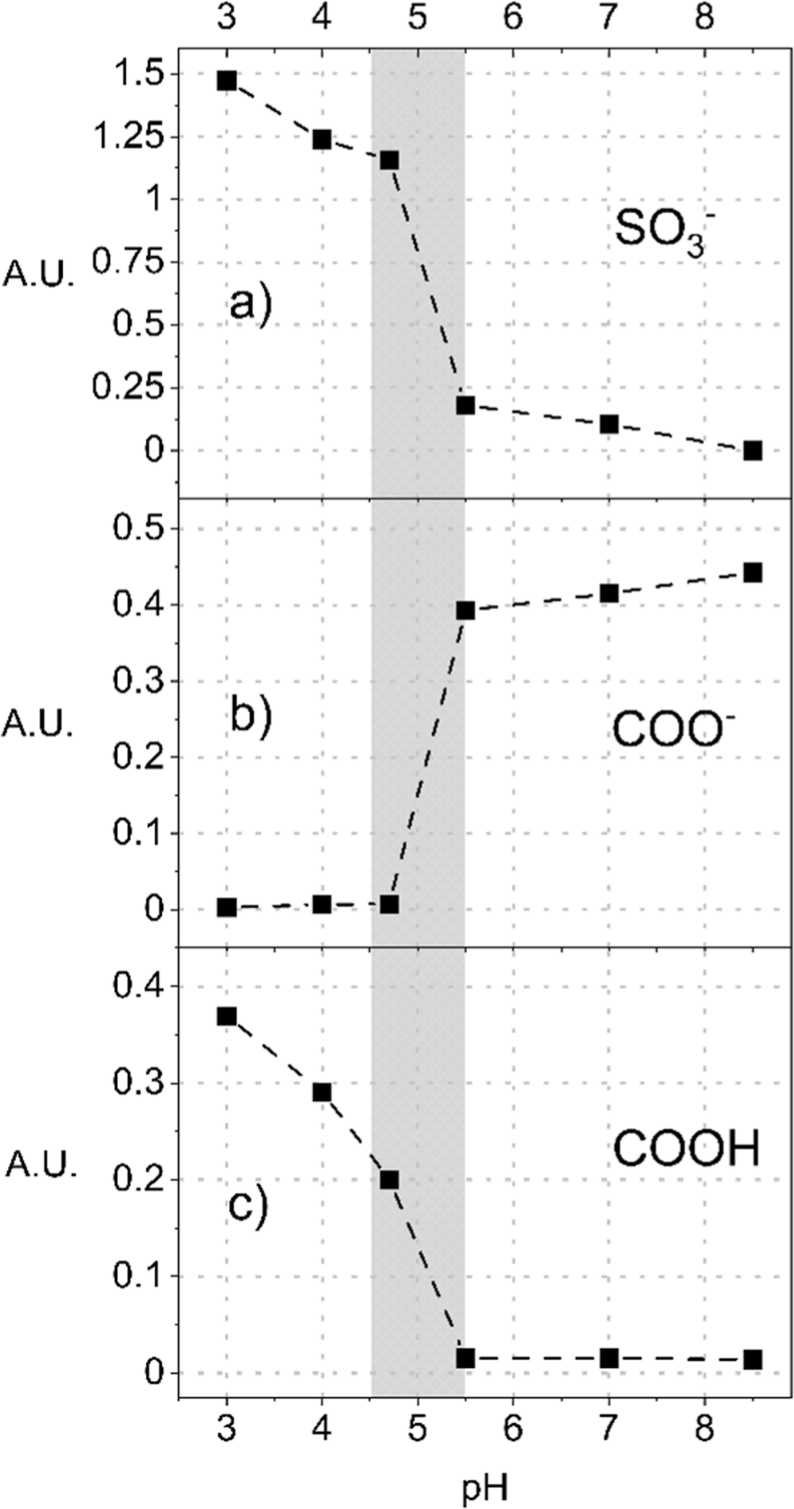
Integral band intensities of the DS sulfate group at 1266 cm^–1^ (a), ionized carboxyl groups at 1611 cm^–1^ (b), and protonated carboxyl groups at 1739 cm^–1^ (c) of pCBMAA vs pH.

The situation is illustrated more quantitatively in Figure 6. The DS band in Figure 6a shows no DS adsorption at pH 8.5, but with decreasing pH, the DS adsorption gradually increases to pH 5.5. Then, between pH 5.5 and 4.7, the DS adsorption increases rapidly, followed by slower adsorption up to pH 3.0. The large increase in adsorption in the pH range of 4.7 to 5.5 correlates well with the charge reversal at pI values 5.6 and 4.5 determined by ZP measurements. The DS adsorption at pH values above the pI, i.e., in the region where pCBMAA should be electrically neutral (based on internal charge equivalency) or negatively charged (based on ZP), is apparently due to a local shift in pH at the interface caused by the strong DS polyanion. This is also evident from the changes in the charge balance of carboxyl groups. Carboxyl groups should be fully ionized at a pH above the pI. However, as shown in Figure 6, within the pH range from 8.5 to 5.5 and in the presence of DS, the intensity of the ionized carboxyl group gradually decreases and the intensity of the DS sulfate band increases. Then, in the pH range of 5.5 to 4.5, there is a sharp decrease in the intensity of the band of the ionized carboxyl groups, together with a sharp increase in the intensity of the DS band. This sharp change is matched by an increase in the intensity of the protonated carboxyl group band. In the pH range from 4.5 to 3.0, the band of the ionized carboxyl groups disappears, but the rise of the DS band and protonated carboxyl groups slowly continues. This reflects the displacement of carboxyl groups from interaction with QA by sulfate groups of DS.

The direct impact of the presence of DS on the balance of carboxyl groups is demonstrated in Figure 7, comparing the intensity of the ionized carboxyl group in the presence and absence of DS.

**Figure 7:**
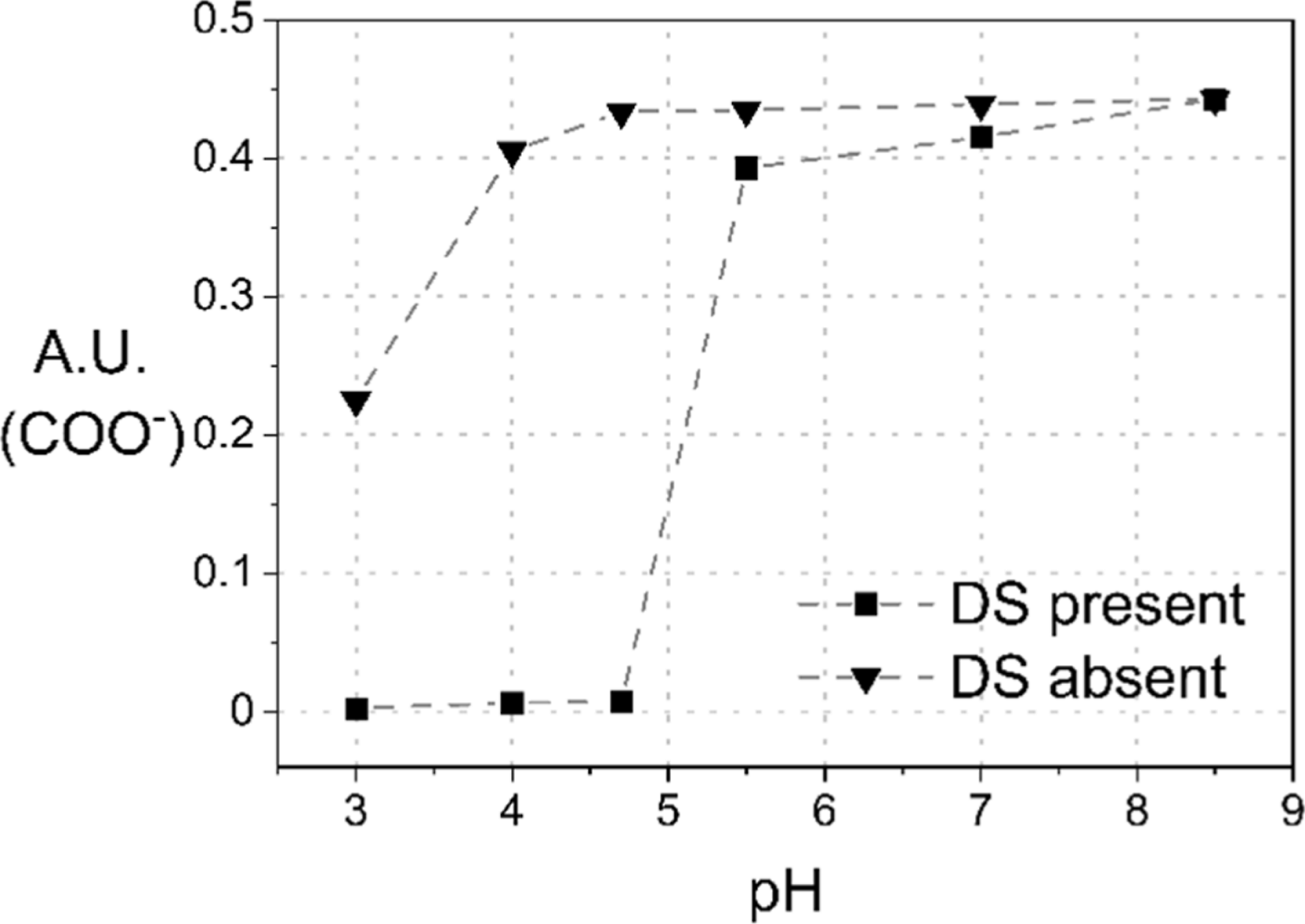
IRRAS integral band intensities of ionized carboxyl groups at 1611 cm^–1^ of pCBMAA in the presence and absence of DS vs pH.

As shown in Figure 8, in the absence of DS, the intensity of the ionized carboxyl group band remains essentially unchanged in the pH range from 8.5 to 4.7, and its decrease in the pH range from 4.7 to 3.0 is significantly smaller than in the presence of DS. Therefore, the carboxyl ionization equilibrium observed by IRRAS is primarily controlled by the adsorbed DS.

**Figure 8:**
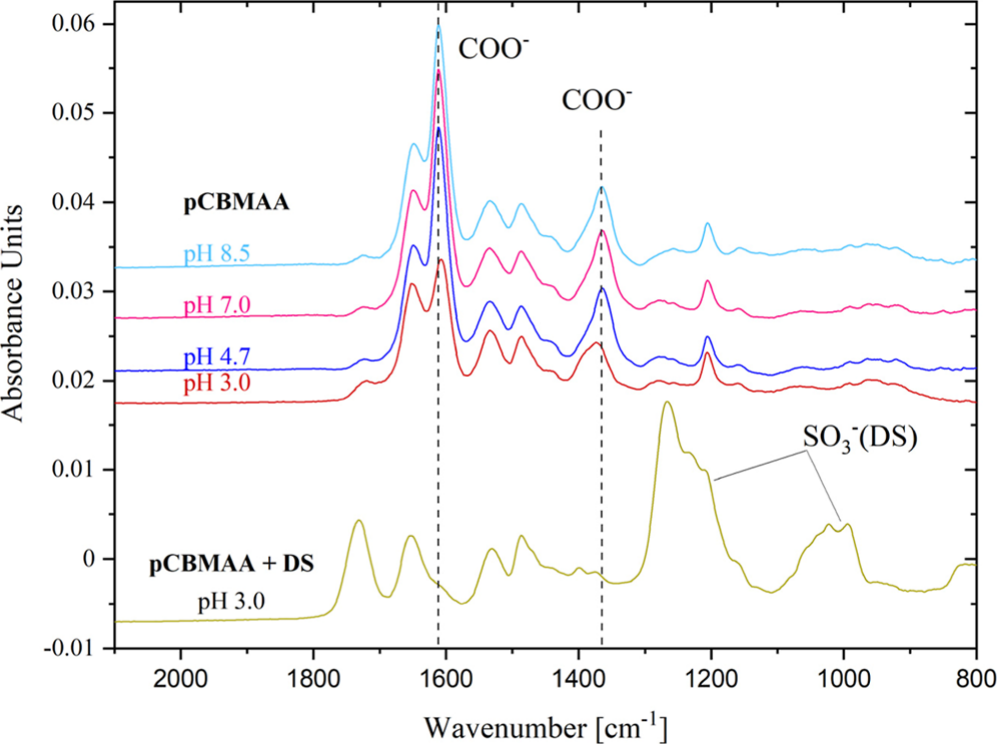
IRRAS spectra of pCBMAA at pH 8.5–3.0 in the absence of DS and the pCBMAA spectrum at pH 3.0 in the presence of DS.

The entire discussion is based on the results obtained using the IRRAS technique, which allows the brushes to be measured in the dry state only. While the DS adsorption at a specified pH can be reliably evaluated using this technique, there is an obvious question about whether IRRAS spectra reflect the true ionization balance of carboxyl groups in the wet state, at least in a qualitative way. The IRRAS spectra were measured as quickly as possible, and the whole procedure, including the quick drying and the spectrum scanning, took no more than 2 min. However, the pH at the time of the IRRAS measurement was not well-defined. The brushes can quickly respond to changes in pH; Figure 9 shows that when the pCBMAA brush with DS adsorbed at pH 3.0 is rinsed briefly with water, the electrostatically attracted DS is completely released and the initial pattern of the ionized and protonated carboxyl groups is restored. The spectra also show no signs of degrafting, i.e., release of the pCBMAA chains after exposure to pH changes and interaction with DS at acidic pH. This is in agreement with our previous findings, which verified very good stability of these brushes,^36,47^ although some other brushes have been reported to degraft upon reswelling.^48,49^

**Figure 9:**
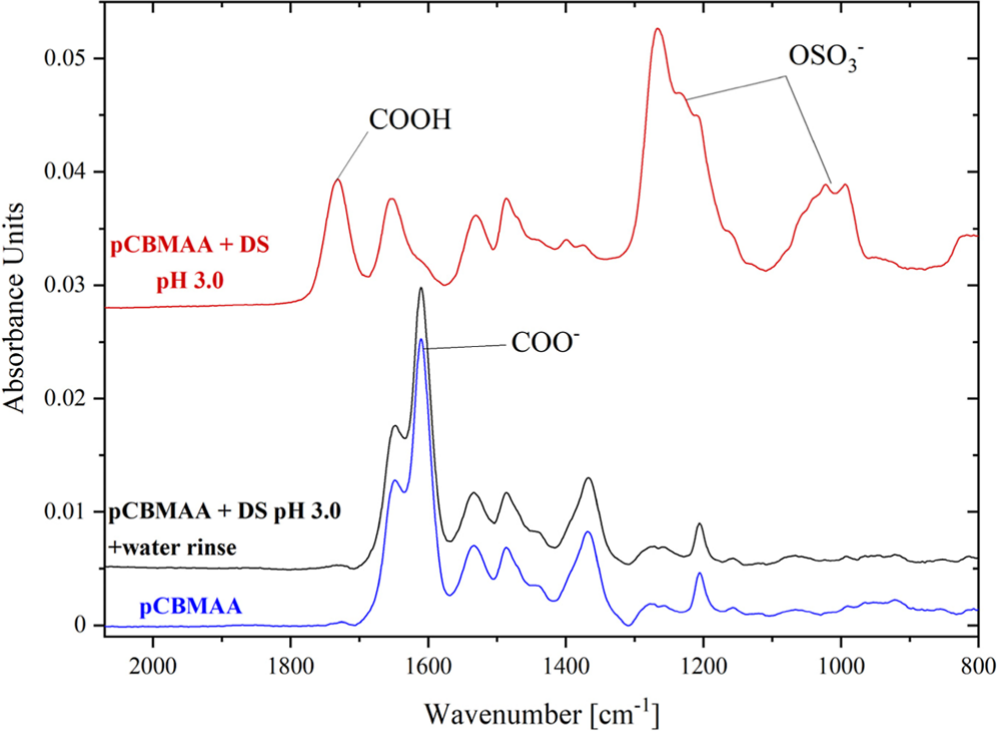
IRRAS spectra of the initial pCBMAA brush, brush with DS adsorbed at pH 3.0, and brush with DS adsorbed at pH 3.0 and then rinsed with water. There is no sign of pCBMAA degrafting upon DS adsorption and desorption.

To address the issue of the COO^–^/COOH balance, we used GAATR spectroscopy. This technique can scan nanolayers sandwiched between two reflective surfaces,^50^ one of which is a gold chip coated with a water-swollen brush and the other is the ATR prism. The gap between these two surfaces is extremely thin, so the interfering water spectrum can be subtracted to obtain the brush spectrum. The main disadvantage is the necessity of physical contact of the sample with the prism, which can damage the sample and make it unusable for further investigation.

Figure 10 shows the GAATR spectra of the pCBMAA brush swollen in water with the pH adjusted from 8.5 to 3.0. At pH 3.0, all carboxyl groups have been protonated, which is a significant difference compared to the IRRAS spectrum (Figures 7 and 8), where the band of the ionized carboxyl groups is also reduced at pH 3.0 but still clearly visible. Therefore, as previously discussed, the changes in the ionization of carboxyl groups observed by IRRAS in the presence of adsorbed DS are primarily driven by the interactions between the pCBMAA brush and DS.

**Figure 10:**
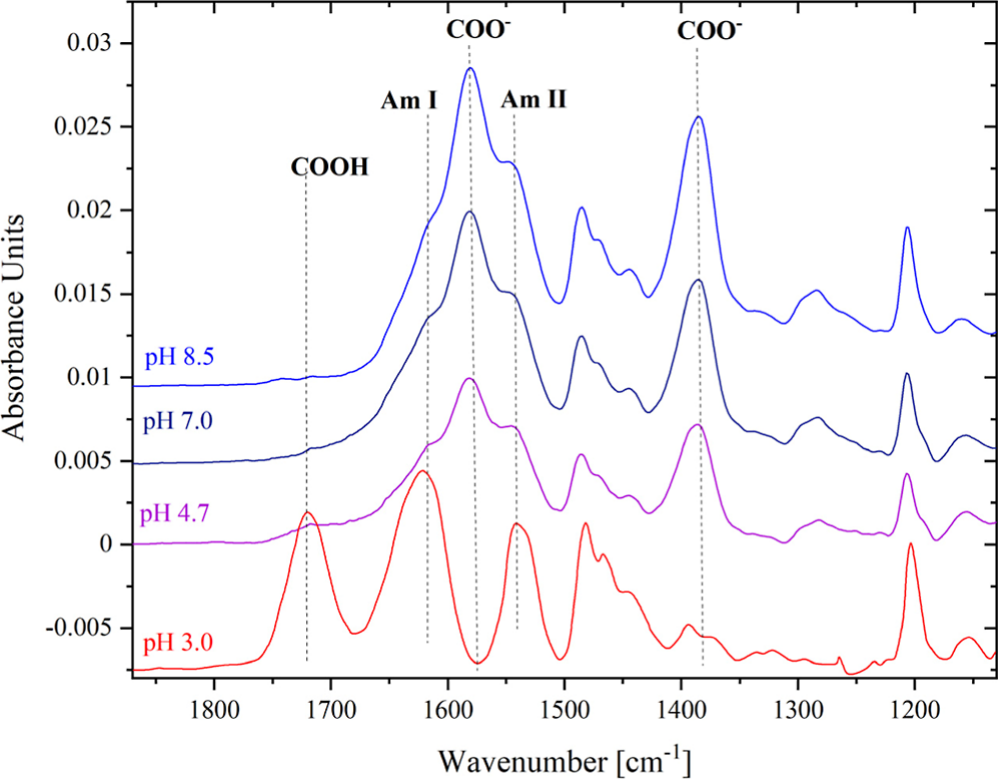
GAATR spectra of the pCBMAA brush scanned under wet conditions at different pH values.

#### 3.2.2 pSBMAA Interactions with DS

The IRRAS spectra in Figure 11, which show the pH dependence of DS adsorption on the pSBMAA brush, reveal a broadened band of the sulfo group of pSBMAA. The difference spectra obtained by subtracting the pSBMAA spectrum reveal weak but well detectable adsorption of DS, which is essentially independent of pH and occurs in both acidic and alkaline regions.

**Figure 11:**
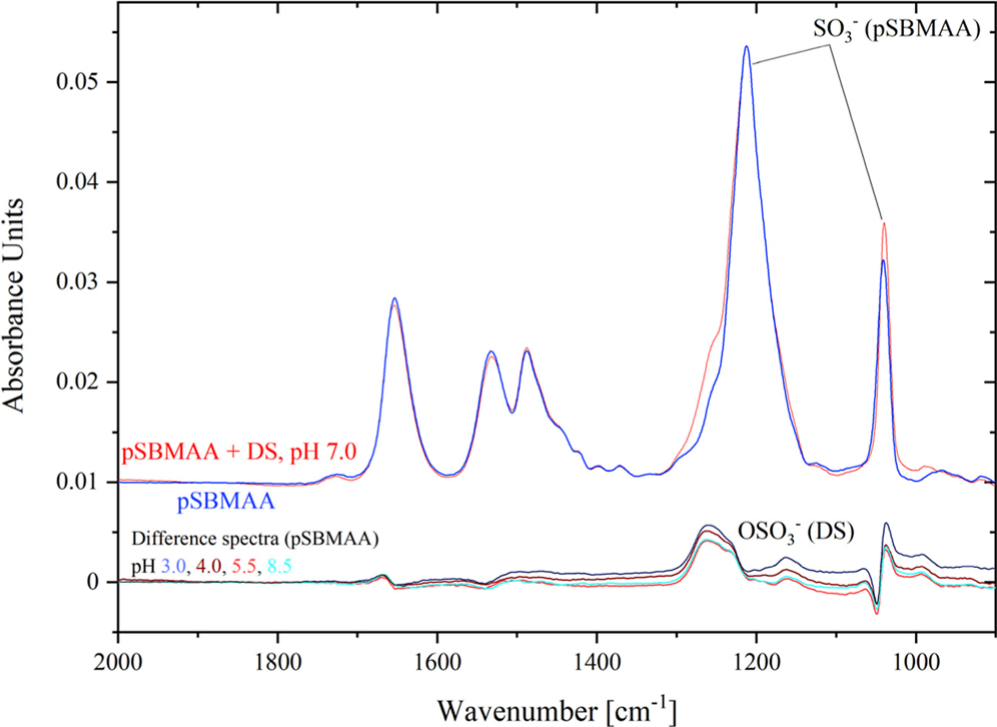
IRRAS spectra of pH dependence of DS adsorption on the pSBMAA brush, indicating a weak DS adsorption at all pH values.

Although the pSBMAA brush is supposed to be electrically neutral, with both sulfo and QA groups permanently charged, the ZP measurement (Figure 2) indicates a pI of 4.0 and a negative potential above its pI. This suggests that even under such conditions, some DS molecules may approach pSBMAA, and its sulfate groups may displace some SBMAA sulfo groups from interacting with the QA groups.

#### 3.2.3 pHPMAA Interaction with DS

The IRRAS spectra shown in Figure 12 indicate no DS adsorption on the pHPMAA brush at any pH. This is consistent with the absence of ionizable groups in pHPMAA and its absence of electrostatic interactions.

**Figure 12:**
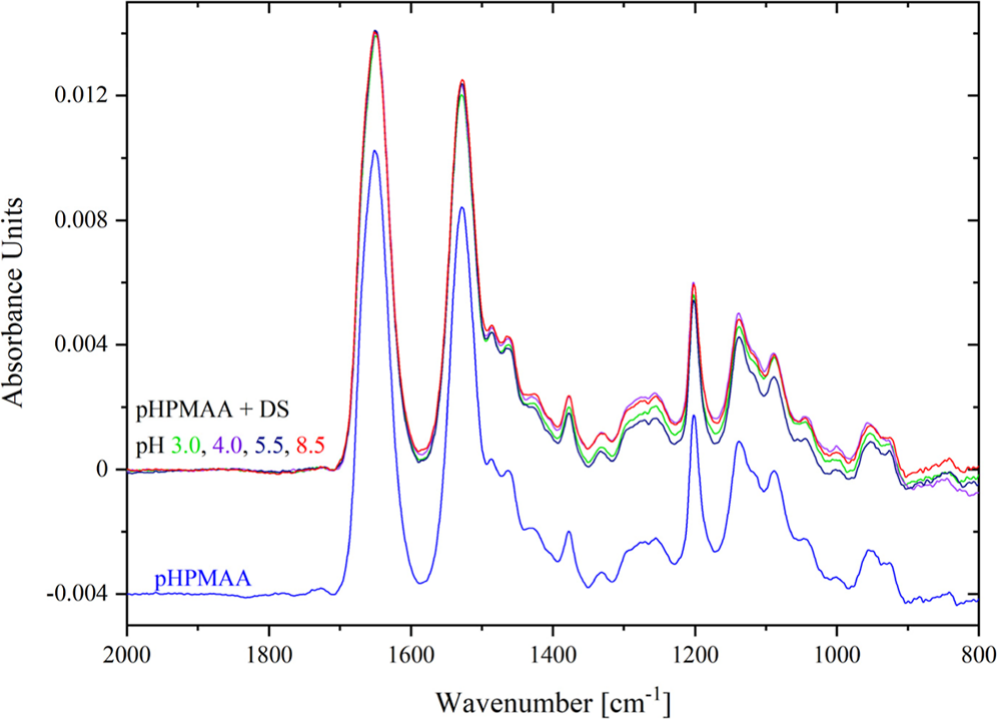
IRRAS spectra of pH dependence of DS interaction on the pHPMAA brush, indicating no DS adsorption at any pH.

### 3.3 pH Dependence of Interactions of pCBMAA, pSBMAA, and pHPMAA Brushes with PTMAEMA

Since the ZP measurement of gold indicates a significant negative potential (−76 mV at pH 6.4, see Table 1), we first checked whether polycation PTMAEMA adsorbs on gold. The spectra in Figure 13a reveal a very weak but discernible PTMAEMA adsorption at all pH values from 8.5 to 3.0. The adsorption of PTMAEMA to gold is apparently not controlled by pH, which is in agreement with its permanent positive charge. However, the high negative ZP of gold does not seem to be effective in attracting larger amounts of PTMAEMA.

**Figure 13:**
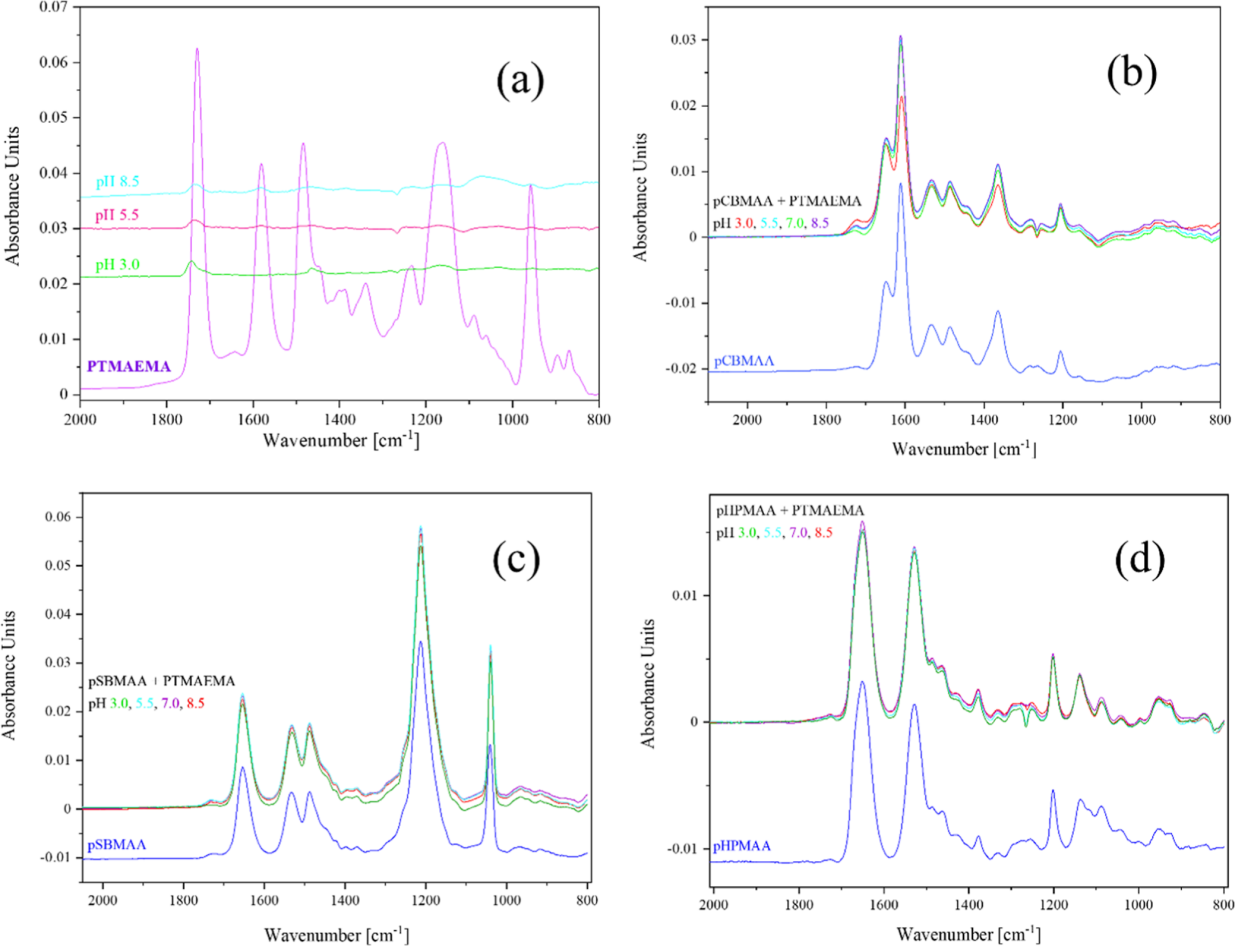
IRRAS spectra of pH dependence of PTMAEMA interaction with gold (a), pCBMAA (b), pSBMAA (c), and pHPMAA (d). There is a weak PTMAEMA adsorption on gold but not on the brushes at any pH.

The IRRAS spectra in Figure 13b show no adsorption of PTMAEMA to the pCBMAA brush at any pH. At pH 3.0, a partial protonation of carboxyl groups was observed (decrease of the carboxylate band), similar to that in the absence of DS (Figure 8). No adsorption of PTMAEMA at pH values above the pI of the pCBMAA brush indicates that there is no effective negative charge on the surface of the pCBMAA brush.

The IRRAS spectra in Figure 13c show no adsorption of PTMAEMA by the pSBMAA brush at any pH. Therefore, this indicates that there is no effective negative charge on the brush surface.

The IRRAS spectra shown in Figure 13d indicate that there is no adsorption of PTMAEMA on the pHPMAA brush at any pH value and that at pH values above the pI, there is no effective negative charge on the brush surface. This is consistent with the fact that pHPMAA does not contain ionizable groups and generally exhibits minimal interactions.

The above findings confirm the general observation that the negative charge indicated by zeta potential determinations does not necessarily correlate with effective surface interactions. The different behavior of the three brushes is schematically illustrated in Figure 14: pCBMAA adsorbs DS strongly below its pI and weakly above its pI; pSBMAA adsorbs DS weakly over the entire pH range; and pHPMAA does not adsorb DS at any pH. None of the brushes adsorb PTMAEMA at any pH.

**Figure 14:**
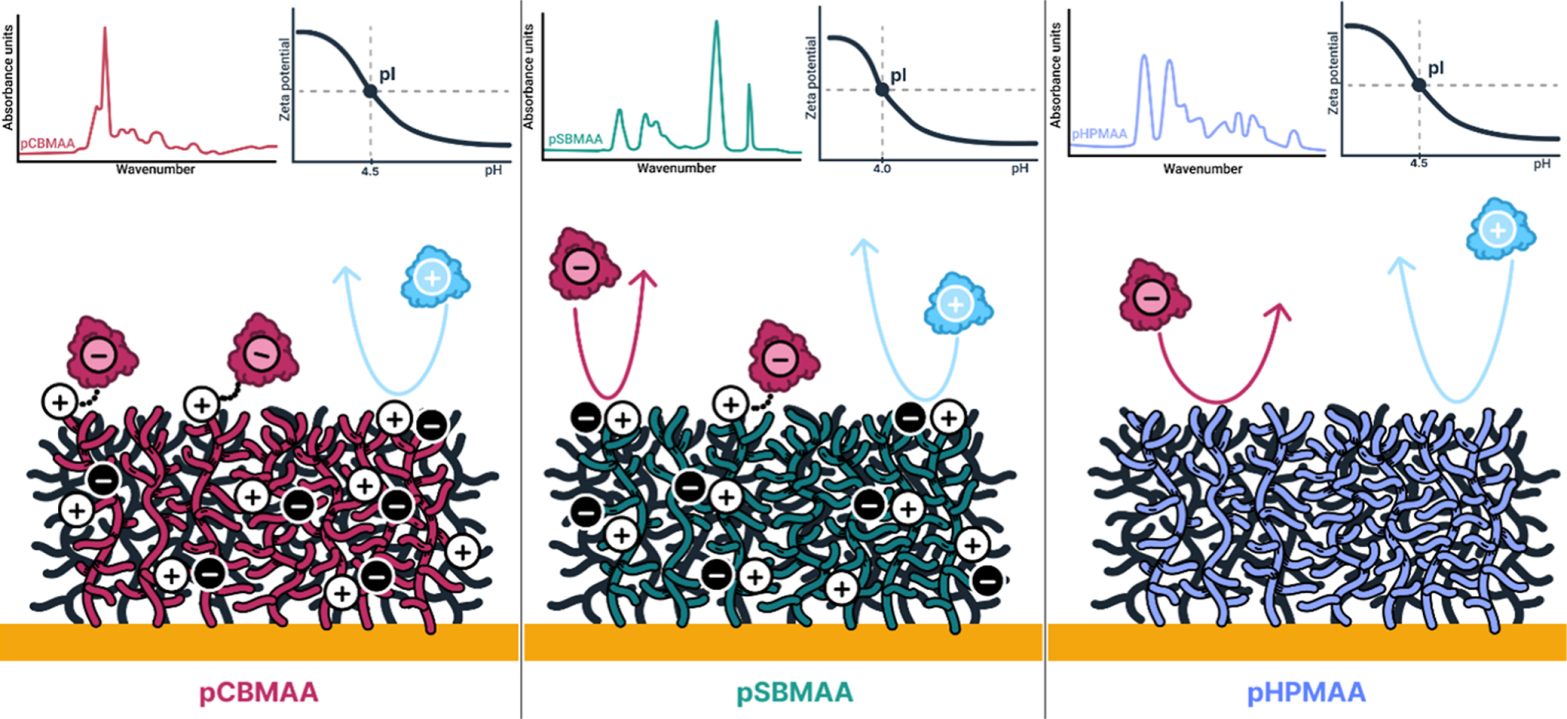
Schematic illustration of surface interactions of pCBMAA, pSBMAA, and pHPMAA brushes with charged macromolecules.

## 4 Conclusions

Two polybetaine brushes on a gold support, poly­(carboxybetaine methacrylamide) (pCBMAA) and poly­(sulfobetaine methacrylamide) (pSBMAA), as well as uncharged poly­[*N*-(2-hydroxypropyl) methacrylamide] (pHPMAA), show negative zeta potentials at pH values above their isoelectric points, contrary to the assumption that these polybetaines and pHPMAA should be electrically neutral. This observation can be explained by the preferential adsorption of hydroxyl anions on the surfaces of these brushes. As the zeta potential plays a crucial role in controlling electrostatic interactions of surfaces, the interactions of the brushes with anionic dextran sulfate (DS) and cationic poly­[(*N*-trimethylammonium)­ethyl methacrylate] (PTMAEMA) were studied using infrared reflection spectroscopy. As expected, DS adsorbs to pCBMAA at pH below its pI, but some DS can adsorb to pCBMAA even at pH values above its isoelectric point and weakly to pSBMAA over a wide pH range. This is apparently due to the displacement of carboxyl and sulfo groups by the interaction of the DS sulfate groups with the quaternary ammonium cation. DS does not adsorb to pHPMAA at any pH despite its zeta potential. The conclusion is supported by the data on the interaction with the cationic PTMAEMA. Although PTMAEMA can weakly adsorb to gold, it does not interact with either pCBMAA, pSBMAA, or pHPMAA brushes on gold at any pH. This confirms the general observation that the negative charge indicated by zeta potential determinations does not effectively control the interactions between the investigated brushes and polyelectrolytes.

## Supplementary Material



## Notes

The fitting parameters of titration data surface zeta potential measurements; AFM images and RMS surface roughness of polymer brushes after incubation at different pH; and thicknesses of polymer brushes swollen in water at different pHs measured by spectroscopic ellipsometry (PDF)
