# Supplementary material for: The Effective Charge of Low-Fouling Polybetaine Brushes
Source: Langmuir. 2025 Jun 10;41(24):15307–18. doi: 10.1021/acs.langmuir.5c00759 (PMC12199473; doi:10.1021/acs.langmuir.5c00759)
Supplement: Supplementary file 1 [file la5c00759_si_001.pdf]

Supporting Information

The Effective Charge of Low-Fouling Polybetaine Brushes

Alina Pilipenco[a,b], Michala Forinová[a,b], Zulfiya Černochová[c], Zdeňka Kolská[d], Ladislav Fekete[a], Hana Vaisocherová-Lísalová[a], Milan Houska[a]*

[a] FZU - Institute of Physics of the Czech Academy of Sciences, Na Slovance 1999/2, 180 00 Prague, Czech Republic

[b] Institute of Physics, Faculty of Mathematics and Physics, Charles University, Ke Karlovu 3, Prague, 121 16, Czech Republic

[c] Institute of Macromolecular Chemistry CAS, Institute of Macromolecular Chemistry CAS, Heyrovského nám. 2, Prague, 16206 Czech Republic

[d] Faculty of Science, J. E. Purkyně University in Ústí nad Labem, Pasteurova 15, Ústí and Labem, 400 96 Czech Republic

* Corresponding author: Milan Houska, FZU - Institute of Physics of the Czech Academy of Sciences, Na Slovance 2, 180 00 Prague, Czech Republic, houska@fzu.cz

Table S1. Surface zeta potential: The fitting parameters of titration data for the pCBMAA, pSBMAA, or pHPMAA brush samples.

Sample	pCBMAA	pSBMAA	pHPMAA
A_1	-61.07±5.57	-42.55±5.21	-48.92±9.10
A_2	37.54±8.57	37.59±13.44	47.05±16.06
P	0.77±0.22	0.537±0.17	-0.45±0.16
Reduced Chi-Square	25.86	10.87	22.52
R-Square (COD)	0.99	0.99	0.99
R-Square	0.98	0.98	0.98

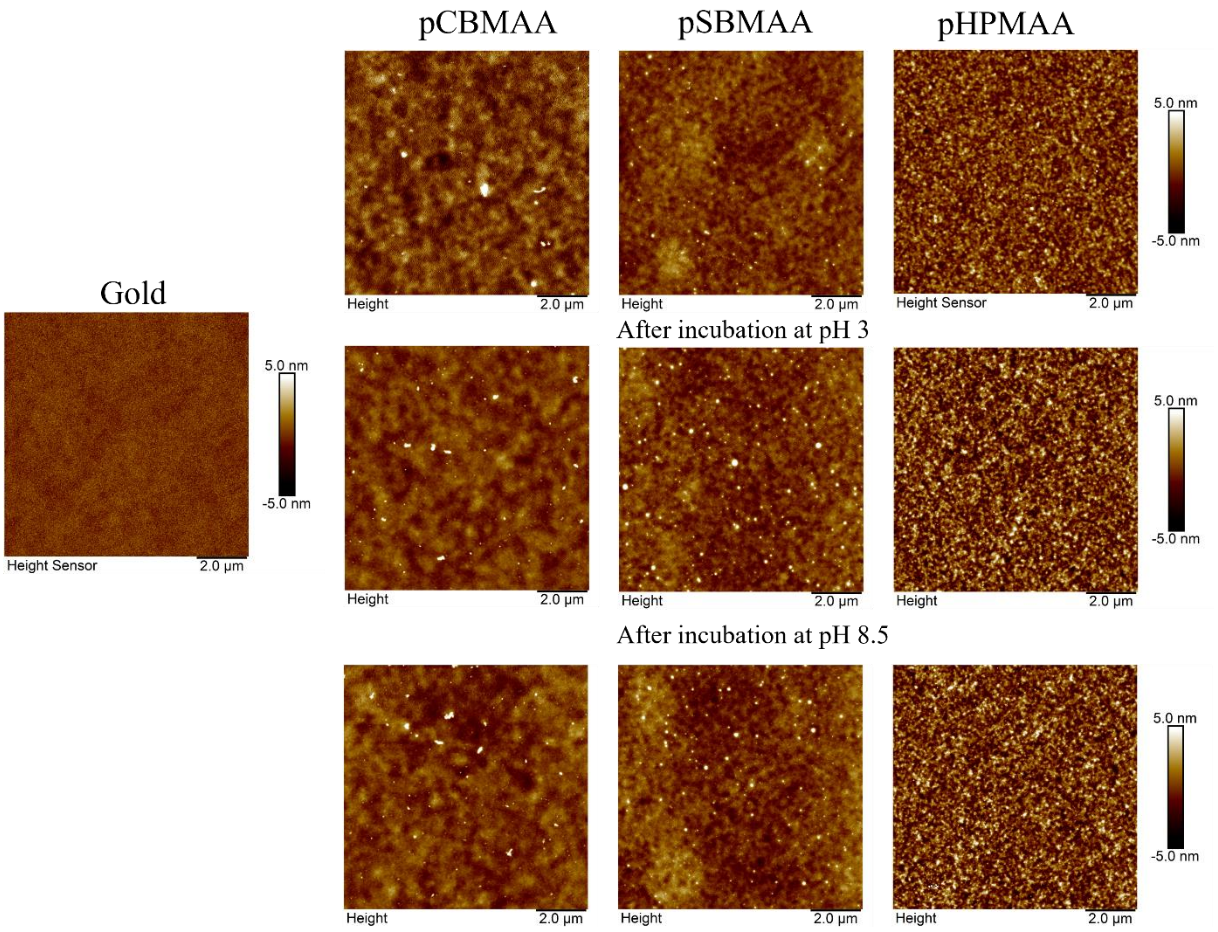

Figure S1. AFM images of pCBMAA, pSBMAA and pHPMAA brushes after incubation in water at pH 3.0 and 8.5.

Table S2. RMS surface roughness of pCBMAA, pSBMAA and pHPMAA brushes after incubation in water at pH 3.0 and 8.5.

Brush	RMS Surface Roughness [nm]		
	Water	pH 3.0	pH 8.5
pCBMAA	0.8	0.7	0.9
pSBMAA	1.2	1.1	1.0
pHPMAA	1.1	1.5	1.5

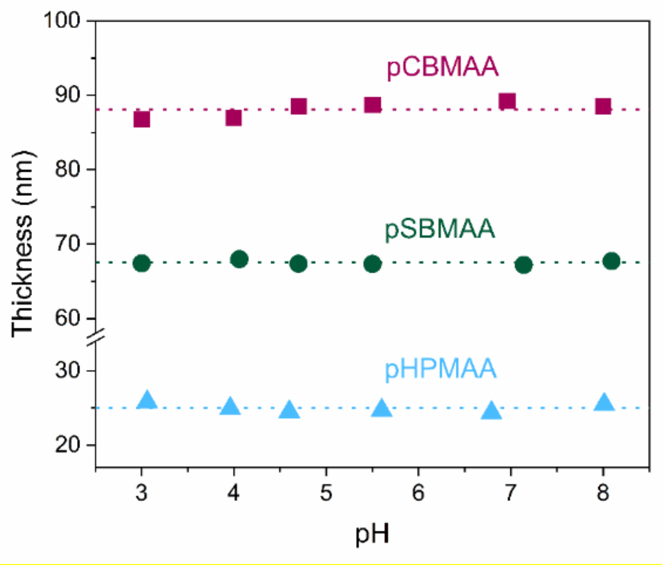

Figure S2. Thicknesses of pCBMAA, pSBMAA and pHPMAA brushes swollen in water at different pH measured by spectroscopic ellipsometry.
